# Aphids: A Model for Polyphenism and Epigenetics

**DOI:** 10.1155/2012/431531

**Published:** 2012-03-21

**Authors:** Dayalan G. Srinivasan, Jennifer A. Brisson

**Affiliations:** ^1^Department of Biological Sciences, Rowan University, Glassboro, NJ 08028, USA; ^2^School of Biological Sciences, University of Nebraska-Lincoln, Lincoln, NE 68588, USA

## Abstract

Environmental conditions can alter the form, function, and behavior of organisms over short and long timescales, and even over generations. Aphid females respond to specific environmental cues by transmitting signals that have the effect of altering the development of their offspring. These epigenetic phenomena have positioned aphids as a model for the study of phenotypic plasticity. The molecular basis for this epigenetic inheritance in aphids and how this type of inheritance system could have evolved are still unanswered questions. With the availability of the pea aphid genome sequence, new genomics technologies, and ongoing genomics projects in aphids, these questions can now be addressed. Here, we review epigenetic phenomena in aphids and recent progress toward elucidating the molecular basis of epigenetics in aphids. The discovery of a functional DNA methylation system, functional small RNA system, and expanded set of chromatin modifying genes provides a platform for analyzing these pathways in the context of aphid plasticity. With these tools and further research, aphids are an emerging model system for studying the molecular epigenetics of polyphenisms.

## 1. Introduction

While the genome has been portrayed as a “blueprint” instructing the development of an adult organism, the articulation of genotype into phenotype is a more complex phenomenon. Context-dependent development and environment-dependent phenotypic variation have been observed for decades [1]. Like the changes in gene expression that intrinsically occur in development, environment can affect gene expression and alter developmental trajectories [2]. If these developmental responses to the environment, and plasticity itself, can increase fitness and are heritable, then morphology, physiology, behavior, or life history strategies can evolve elements of adaptive phenotypic plasticity [1, 3]. This can result in the production of continuous or discrete phenotypic variation (polyphenism). The possibility for nongenetic heritable effects of environment on development raises doubts about the “blueprint” view of the genome [4].

Waddington originally defined “epigenetics” as the study of phenomena that act to produce phenotype from genotype all within in a framework of evolutionary biology [5–7]. Waddington's view of epigenetics now largely encompasses the fields of developmental biology and evolutionary developmental biology, which describe, in part, how patterns of gene expression change during ontogeny and through evolution [8]. The modern field of epigenetics examines how patterns of gene expression, instructed by extrinsic biotic or abiotic factors, can be passed to offspring through means other than the inheritance of DNA sequence. Examples of inherited epigenetic phenomena include stable cell fate specification during pluripotent stem cell divisions, dosage compensation and X chromosome inactivation, imprinting, and position effect variegation in *Drosophila* [9]. Models for seemingly disparate phenomena have converged on common mechanisms for establishing heritable gene expression patterns: changes in chromatin architecture due to the effects of DNA methylation, small RNAs, and chromatin modifying enzymes [10, 11].

## 2. Predictive Adaptive Developmental Plasticity through the Aphid Life Cycle

Aphids, soft-bodied insects that feed on the phloem sap of plants, have long been a model for studying the causes and consequences of phenotypic plasticity. They exhibit both a wing polyphenism (consisting of winged and unwinged females) and a reproductive polyphenism (consisting of asexual and sexual individuals). The production of alternative morphs by genetically identical individuals by definition involves epigenetic mechanisms. Here, we describe these two polyphenisms within the context of the aphid life cycle. We then discuss the environmental cues that trigger the polyphenisms. Finally, we discuss what is known about epigenetic mechanisms in the pea aphid.

The life cycle of a model aphid species, the pea aphid *Acyrthosiphon pisum*, begins as a “foundress”—a female aphid that hatches in the spring from an overwintering egg. The foundress produces, via live birth (viviparity), a population of female unwinged aphids through asexual reproduction (apomictic parthenogenesis) that continues to reproduce asexually over several generations. This population is genetically identical, aside from spontaneous mutations [12], and lacks males during the spring and summer months. Environmental factors such as high aphid density, host plant quality, and predation can induce unwinged females to produce winged offspring. Winged asexual females disperse and colonize new host plants, founding new colonies via parthenogenesis. The parthenogenetic production of winged and unwinged female aphids continues during the spring and summer.

In fall, a change from asexual to sexual reproductive modes occurs. Asexual females sense the changing photoperiod and temperature and respond by parthenogenetically producing sexual females and males. Males are produced genetically by the loss of one X chromosome during parthenogenetic oocyte division and can be winged or unwinged. Since only sperm containing an X chromosome are viable, sexual females lay only female eggs on the host plant. The egg must “overwinter” for three to four months at cold temperatures in order to complete development and hatch as a foundress in the spring [13, 14]. Other aphid species switch host plants, produce winged sexual females or produce males earlier than pea aphids. These adaptations (sexual versus asexual, winged versus unwinged) have evolved in response to environmental changes that are predictable (seasons) and unpredictable but common (population density, host plant quality, and predation).

## 3. Experimental Evidence for Epigenetic Phenomena in Aphids

The wing and reproductive polyphenisms are examples of how the maternal environment affects the development of the offspring as a “predictive adaptive response” [15]. Several groups have described the triggering environmental cues and aphid responses. Though the cues differ for the reproductive and wing polyphenisms, the developmental response for both is separated by at least one generation from the triggering cue. Additionally, the resulting morphs are discrete forms and not simply continuous differences along a phenotypic gradient. This binary phenotypic output from an inductive signal gives the aphid experimental system an advantage for studying the epigenetic contribution to phenotypic plasticity.

### 3.1. Induction of Winged Aphids

Winged offspring can be induced by tactile stimulation of unwinged asexual aphids, either by interactions with other aphids, interactions with nonpredator insects, or experimental stimulation [16–18]. Unwinged mothers produce both unwinged and winged offspring; winged aphid mothers rarely produce winged offspring [16]. Other factors, such as the age of the mothers and temperature, can also modulate the degree of wing induction [18, 19]. The production of winged offspring can also be induced by the presence of aphid predators [20–22]. However, this effect may be driven by increased aphid walking, and thus increased inter-aphid interactions, in response to predator presence [23]. The environmental changes listed above are unpredictable but generally common, and aphids facultatively express the wing phenotype to limit predation and competition for resources.

In some aphids, wing induction occurs prenatally [24] while other species can be induced postnatally [16]. In prenatal determination, the environmental cue perceived by the mother must be transmitted to its embryos *in utero*, and the daughter embryos respond to this maternal signal. The precise nature of this maternal signal or its response is not known, though some studies implicate the juvenile hormone (JH) pathway (but see [16]). However, wing development itself does not occur until the second to third larval stage and is accompanied by the development of wing musculature, increased sclerotization of the cuticle, changes in eyes, antennal sensory rhinaria, and reproductive output [25, 26]. Thus, several days and presumably several rounds of cell division separate induction and resulting developmental response.

### 3.2. Induction of Sexual Aphids

The production of sexual morphs and the resulting overwintering egg coincides with predictable, seasonal changes in photoperiod and temperature. Sexual aphid morphs are observed in temperate zones during the fall and winter but not in the spring or summer, and aphids were the first animals shown to respond to changes in photoperiod [27]. Later studies defined the lengths of light and dark phases necessary for the induction of sexual aphids (reviewed in [28]). An embryo that developed under experimentally controlled long-day “summer” conditions (16 hours of light, 8 hours of darkness), and shifted to short day “fall/winter” conditions (12 hours light, 12 hours darkness) upon birth, can produce sexuals-producing mothers that consequently give birth to sexual offspring [28]. Experimental manipulations of temperature can modulate the degree of sexual morph production [29], and high temperatures can override the effect of short days on sexual induction [29, 30]. Based on the timing of sexual offspring birth, determination of embryos destined to become sexual morphs is thought to occur after embryonic germ cell cluster formation and migration, roughly corresponding to stage 17 of asexual embryo development [13, 31]. Induction of sexual-producing aphids and their sexual offspring requires at least 10 consecutive days of fall/winter conditions [32], which appears to prevent aphids born prior to the vernal equinox from undergoing sexual induction. Some strains of aphids also produce sexual aphids followed by asexual females, possibly to hedge bets against a harsh winter and the lack of host plants [33, 34].

Similar to winged aphid induction, the induction of sexual aphids is a complex process that involves multiple tissues and extends over several days of development. Though external morphological differences between asexual and sexual females are few, the difference in internal morphology is striking. Aphid ovaries consist of 12–16 ovarioles, each of which contains germ cells housed in an anterior germarium. In sexual ovaries, germaria are connected to oocytes [31, 35]. The sexual haploid oocyte will fill with yolk contributed by nurse cells in the germarium, grow in size, and pass through the uterus to undergo fertilization. However, in asexual ovaries, the germarium is connected to a posterior string of successively older asexual embryos progressing through development, from one-celled embryos to fully developed embryos ready for parturition.

Both aphid polyphenisms are examples of the maternal epigenetic determination of offspring phenotype. The maternal inducing signal, received by the offspring as embryos, is translated into an expansive suite of developmental changes well after birth. Over 90 years ago, Ewing [36] reviewed several studies on wing induction and postulated a transgenerational “physiological inheritance” that is “not dependent on the germplasm (or at least the chromosomes) but which modifies the expression of somatic characters.” Sutherland [37] also hypothesized a nongenetic “intrinsic factor” that delayed production of winged offspring from mothers born early from winged grandmothers. The transgenerational response to changing environmental conditions in aphids in some cases may involve juvenile hormone (JH). Application of JH or JH analogs to aphid mothers can prevent sexual induction under fall/winter conditions [38, 39]. Neurosecretory cells within the mother's brain likely perceive light and dark and transduce the photoperiod signal to the progeny directly or indirectly through JH [40, 41]. Thus, in the reproductive polyphenism, this “physiological inheritance” may be due to maternal hormonal signals that establish heritable epigenetic information that sets gene expression patterns in the developing embryo. Below, we discuss how genomics technologies and bioinformatics have invigorated investigation of the molecular basis of this epigenetic phenomenon.

## 4. The Aphid Genome: A Model for Plasticity

The genome of the pea aphid *A. pisum* is distinctive among insect and even animal genomes for several reasons [42]. With its large size (~517 Mbp) and large number of predicted genes (~35,000 genes, many well-supported by homology, EST, or RNA-seq data), the pea aphid possesses one of the largest gene repertoires among animals, rivaling that of *Daphnia pulex*, another polyphenic arthropod [43]. Repetitive elements (REs) account for a large fraction of the assembled genome (38%) [42, 44]. The large number of genes is due to a large number of gene duplications: 2,459 gene families of various functions have undergone duplication, with many families containing more than 5 paralogs. Indeed, paralogs account for nearly half of the total aphid genes, similar to that of *Daphnia* [43]. Notable among these are duplications of genes involved in DNA methylation, small RNA pathway, and chromatin modifications and remodeling (discussed in detail below). Furthermore, the aphid genome has the lowest G/C content among sequenced insects at 29.6%. The pea aphid community now has an impressive set of genomic data and tools: a draft genome sequence, expressed sequence tags (EST), full-length cDNA sequences, microarrays, and RNAi [42, 45–60].

This genome information can be leveraged toward understanding the basis of aphid plasticity and the role of epigenetics in that plasticity. For example, aphid-specific gene duplications may have facilitated the evolution of developmental plasticity, as greater phenotypic space can be explored through the differential expression of diverged paralogs in response to environmental variation. Indeed, reports of differential paralogous gene expression between different aphid morphs lend support to this hypothesis [51, 61–65]. The molecular basis for the differential expression of aphid paralogs is thus far unknown. We speculate that, in a manner similar to other arthropods [66], environmentally sensitive expression of maternal hormones helps establish heritable patterns of chromatin architecture in the embryo that affect gene expression patterns during development. This could involve DNA methylation, which can regulate gene expression in arthropods [67, 68], small RNAs, and chromatin modifications. Below, we discuss recent results lending support to a functional epigenetic system in aphids that may underlie polyphenic aphid development.

## 5. DNA Methylation

Several epigenetic processes rely on DNA methylation, which involves the addition of a methyl group (–CH_3_) to the 5-carbon of cytosine in genomic DNA to form 5-methylcytosine. Methylation modifications are most commonly found on cytosines at CG dinucleotides, resulting in a symmetrical double-stranded pattern. They are less commonly found in a CHG or CHH context, where H = A, G, or T [69]. These methyl groups act as a “memory” at particular genes and function during the normal growth and differentiation of many organisms [70, 71]. DNA methylation can negatively affect transcription by either physically interfering with the binding of proteins that activate transcription, or recruiting other proteins that affect chromatin structure (see Chromatin Remodeling section). They also silence the activity of transposons and inactive genes [72].

In aphids, DNA methylation was originally observed at the E4 esterase gene in insecticide-resistant green peach aphids, *Myzus persicae* [73–75]. Contrary to the generally understood role of DNA methylation in negatively regulating transcription, the E4 esterase gene was only expressed when it was methylated [76]. At the time, few studies had investigated the functional consequences of the observed low levels of methylation in insects [67]; thus, few conclusions could be made about the role of methylation in vertebrates versus invertebrates. Mandrioli and Borsatti [77] reported the presence of DNA methylation in the heterochromatic regions of pea aphid DNA, although they did not identify specific regions that were methylated.

**Figure 1:**
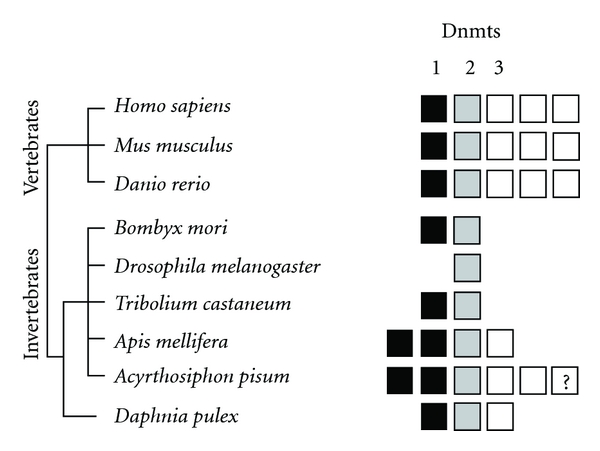
Vertebrates and invertebrates vary in Dnmt subfamily enzyme copy number. The number of boxes in each color (black, grey, white) indicates the number of paralogs of each type of Dnmt.

DNA methyltransferases (Dnmts) are the enzymes that add methyl groups to nucleotides in DNA, using S-adenosyl methionine as the methyl donor. Animals use three classes of Dnmts [69, 78]. Dnmt1 acts as a maintenance methyltransferase, attaching methyl tags to newly synthesized DNA strands; Dnmt3 typically methylates DNA *de novo*; Dntmt2, an RNA cytosine methyltransferase, is no longer considered a true DNA methyltransferase [79, 80]. However, current evidence suggests that all three active Dnmts (Dnmt1, Dnmt3a, and Dnmt3b) may be involved in the maintenance of DNA methylation [81]. Considerable variation across taxa exists as to the presence or absence of each category of Dnmt [82]. For example, the honey bee (*Apis mellifera*) has two copies of Dnmt1, one of Dnmt2 and one of Dnmt3 [83], while *C. elegans* has lost all Dnmts and seems to lack DNA methylation [70, 84] (Figure 1). Clearly, some organisms develop and reproduce successfully without methylation enzymes and thus without methylated DNA.

The previous reports of methylated aphid DNA indicated the presence of Dnmts in the aphid genome. However, given variation among taxa in Dnmt occurrence, it was not obvious *a priori* that an aphid genome would contain all of the DNA methylation enzymes. By searching the pea aphid genome sequence [42], Walsh et al. [85] found two copies of Dnmt1, a Dnmt2 a Dnmt3, and a gene distantly related to the other Dnmts that they called Dnmt3X. Dnmt3X lacks key amino acids thought to be necessary for Dnmt function. It may, therefore, be a pseudogene. Additional proteins involved in DNA methylation are present in the pea aphid genome: the methylated-CpG binding proteins MECP2 (one copy) and NP95 (three copies), and Dnmt1 associated protein that associates with Dnmt1 to recruit histone deacetylases [85, 86]. Walsh et al. [85] also quantified overall methylcytosine levels, finding that 0.69% (±0.25%) of all of the cytosines were methylated. This low percentage closely matches the low methylation levels observed in other insect genomes [82]. Further, twelve pea aphid genes are methylated in their coding regions, but not in their introns [85]. Three of those genes are juvenile hormone (JH) associated genes, chosen for analysis because JH has previously been shown to be involved in phenotypic plasticity in aphids [28]. Further investigation of the gene for JH binding protein revealed one methylated site that had a marginally significant higher level of methylation in winged relative to wingless asexual females [85]. Overall, these data indicated that the pea aphid has a functional DNA methylation system.

## 6. Aphid Genome Methylation Patterns

With the pea aphid genome sequence, patterns of DNA methylation could be investigated using an indirect measure that utilizes the observed versus expected levels of CpG methylation (CpG_O/E_). This method is based on the fact that methylated cytosines are hypermutable, resulting in a loss of CpGs in methylated regions. Regions of DNA with low CpG_O/E_ are inferred to have been historically methylated and thus are considered areas of dense methylation [87].

Walsh et al. [85] used this method to examine the coding regions of all predicted genes in the pea aphid genome. The resulting histogram of gene frequency by CpG_O/E_ exhibited a clear bimodal distribution, indicating two gene classes: genes with and without a history of DNA methylation. This same pattern was observed in another polyphenic species, the honey bee, whereas it was not observed in nonpolyphenic species like the red flour beetle (*Tribolium castaneum*), *Anopheles gambiae, *and *Drosophila melanogaster* [88]. These data began to approach the intriguing question of whether methylation levels associate with aphid alternative phenotypes, but to take this question a step further required gene expression data.

Brisson et al. [89] used a pea aphid microarray to identify significantly differentially expressed (DE) genes between fourth instars and adults, males and females, and wing morphs within each sex (wing morphology in asexual females is polyphenic, while in males it is genetically determined). Using these data, Hunt et al. [90] asked whether gene methylation density associated with patterns of DE genes among the different phenotypic groups. Overall, genes with condition-specific expression (i.e., genes with DE among categories) showed higher CpG_O/E_ levels than genes that were more ubiquitously expressed. They concluded that morph-biased genes have sparse levels of methylation while non-morph-biased genes have dense levels of methylation. In a similar study, Elango et al. [88] showed that genes with DE between honey bee queens and workers had higher CpG_O/E_ levels. These studies, along with others [68, 91], suggest that ubiquitously expressed genes in insects are the most likely to be densely methylated.

What are the gene categories with low and high CpG_O/E_ values? The highly methylated class includes gene ontology (GO) terms associated with general organismic functions such as metabolic processes. In contrast, genes with sparser methylation encompass a wider variety of functions such as signal transduction, cognition, and behavior [90]. Given the putative role for methylation in alternative morphologies, these patterns are counterintuitive since morph-biased genes would be presumed to be the most highly methylated. One way to reconcile this contradiction is to modify the hypothesis: if morph-biased genes have sparser CpG methylation, their CpG sites are available for the action of *de novo* methylation. These genes could then acquire differential methylation states, and thus different expression states, on a generation-by-generation basis, induced by relevant environmental circumstances. In support of this, RNAi of the Dnmt3 *de novo* methyltransferase in honey bees led to changes in reproductive morph specification [92].

These previous studies relied on indirect measures of methylation specifically focused on methylation at CG dinucleotides. A catalog of all base positions in the genome that exhibit methylation, known as a “methylome,” would allow for global comparative analyses of DNA methylation. This has been achieved in other organisms (e.g., [91, 93, 94]), and indeed a pea aphid methylome is currently being pursued (O. Edwards, D. Tagu, J. A. B., S. Jaubert-Possamai, unpublished data). With the methylome, it will be possible to answer the following questions: Are there differences in CG, CHG, or CHH methylation patterns between winged and wingless or sexual and asexual females? If so, what specific genes exhibit methylation differences between morphs? Does methylation associate with alternative splicing? Does methylation have a role in regulating the abundant paralogs in the pea aphid genome? Does methylation correlate with expression levels?

## 7. Chromatin Modification and Remodeling Pathway

The production of a cell fate relies on stable gene expression patterns specified by intrinsic and/or external factors during development. Current models propose that DNA methylation and chromatin architecture set stable, yet modifiable, patterns of gene expression. An array of different DNA-bound proteins, largely consisting of histones, acts in concert to create higher-order structures that alter chromatin shape from local to global scales. Histones H2A, H2B, H3, and H4 form an octamer on which DNA is wrapped, forming a structure known as a nucleosome, that can make DNA locally inaccessible to DNA-binding factors. Histone tails extend from the core octamer and are available for modification such as acetylation, ADP ribosylation, methylation, phosphorylation, SUMOylation, and ubiquitylation. These modifications affect local chromatin function by adjusting its accessibility and attractiveness to regulatory complexes [95]. Variant histones can replace core octamer subunits, endowing the local chromatin environment with unique structural and functional properties [96]. Nucleosomes themselves can be repositioned to allow local access to DNA by nucleosome remodeling complexes [97]. This large array of activities is thought collectively to establish a “code” of chromatin characteristics, which reflects the functional and structural state of the underlying chromosomal DNA. Histone modifications, nucleosome remodeling, DNA methylation, and even small RNA pathways may be functionally linked and interdependent in a context-dependent manner [98–100].

Increasing evidence shows that a simple model of “open” and “closed” chromatin is insufficient to explain functional and structural differences among different regions of the genome. Instead, chromatin structure can be viewed as a composite of structural and functional domains with unique combinations of histone post-translational modifications, DNA methylation patterns, variant histone members, nucleosome position and chromosome territory within the nucleus [101, 102]. Chromatin structure is maintained across mitotic divisions, although theoretical and experimental evidences have not yet converged on a mechanism for that transmission.

A survey of aphid chromatin genes is the first step in understanding how heritable chromatin structure may be associated with aphid polyphenisms. The current draft of the aphid genome indicates expansions of antagonistic chromatin modifying and remodeling pathways [61]. Histones and histone variants are conserved in the aphid genome at numbers similar to *Drosophila melanogaster*, though histone variants such as *Cenp-A* and protamines appear absent [61]. The major chromatin remodeling complexes (SWI/SNF, CHD1, ISWI, and NURD) are represented in the aphid genome. The most striking observation is that expansions of gene families involved in histone acetylation are mirrored by expansions of genes involved in histone deacetylation. A similar situation is seen for genes involved in histone methylation and histone demethylation [61, 103]. Since the effect of acetylation and methylation on chromatin state and gene expression is context-dependent, these multiple antagonistic activities could contribute towards a complex regulation of chromatin state in aphids.

Evidence thus far for morph-associated chromatin architecture in aphids is in its early stages. The holocentric structure of aphid chromosomes (which presumably have diffuse kinetochores) could have effects on higher-order chromatin structure. Stainings of pea aphid chromatin detected several histone modifications, such as methylation of histone H3 on lysine 4 and lysine 9 [61, 77, 104]. In particular, largely overlapping differential histone methylation of these residues was observed in specific regions of chromatin [61]. Duplications of antagonistic histone modifying genes could be interpreted as a “need” for a balance of chromatin modifying activities. Alternatively, these duplications could be merely coincident with the general level of gene duplication in the aphid genome and may not have biological relevance for any specific trait. Chromatin immunoprecipitation (ChIP), expression analysis, and evolutionary analysis of these genes should help distinguish between these hypotheses. Additionally, next-generation sequencing technologies can be used to survey morph-specific chromatin modifications [105].

## 8. Small RNA Pathway

Work over the last 15 years has implicated small noncoding RNAs as a layer of epigenetic control. Small RNAs direct the transcriptional or post-transcriptional repression of gene activity in a gene-specific manner. Classes of small noncoding RNAs include endogenous microRNAs (miRNAs); exogenous and endogenous short interfering RNAs (siRNAs and esiRNAs); and Piwi protein-associated small RNAs (piRNAs) [106–108]. This dizzying array of small RNAs is generated by transcription either of endogenous miRNA- and siRNA-encoding genes, or of repetitive elements, transposons and noncoding regions [109]. These classes differ in their biogenesis, processing, function, and partner proteins [110]. Here, we discuss progress in studying aphid miRNA and piRNA pathways.

**Figure 2:**
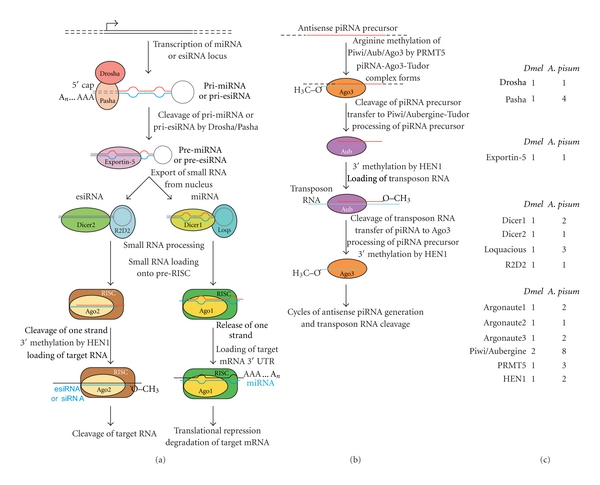
Small RNA pathways are conserved between *Drosophila melanogaster* and *A. pisum*. (a) The esiRNA and siRNA pathway is initiated typically with nearly perfectly complementary dsRNA produced endogenously or introduced exogenously, respectively. miRNAs are endogenously transcribed and processed by a parallel pathway in *Drosophila*, arises from imperfectly complementary dsRNAs, and repress translation of endogenous genes. (b) piRNAs are generated from piRNA clusters in the genome and are processed by a different set of Argonaute family proteins to repress transposon activity. (c) Comparison of small RNA pathway gene copy number between *D. melanogaster* and *A. pisum* reveals aphid-specific duplications. *Dmel*: *D. melanogaster*.

The miRNA and siRNA pathways provide animals and plants a means of attenuating the activities of viruses and selfish genetic elements [111]. Additionally, miRNAs post-transcriptionally regulate the expression of many endogenous genes [112, 113]. Primary miRNA transcripts in the form of a stem-loop are processed by the Drosha/Pasha complex [114] into pre-miRNAs which are exported from the nucleus via Exportin 5 [115–117]. The Dicer1/Loquacious complex then pares the pre-miRNA down to a 21-nucleotide miRNA duplex [118–120]. Mature miRNAs or endogenous siRNAs are then loaded onto a RNA-induced silencing complex (RISC), which contains an Argonaute (Ago) family member protein, of which there are five in *Drosophila* (Ago1–3, Piwi and Aubergine) [121]. In *Drosophila*, Ago1-containing RISCs bind miRNAs while Ago2 RISCs contain siRNAs [122]. One strand of the duplex is retained in this complex as the single-strand miRNA or “guide” siRNA [123, 124]. RISC facilitates annealing of the single-strand miRNA to 3′ UTRs of target mRNAs to either block protein translation and promote target mRNA degradation [109], or, if the miRNA is nearly fully complementary to the target, direct cleavage of the target mRNA by RISC, similar to a siRNA (Figure 2).

In *Drosophila*, *Ago3* and the germline-specific *Piwi* and *Aubergine* Argonaute subfamily members associate with Piwi-associated piRNAs [125–127]. This class of small RNAs arises from “piRNA clusters” in heterochromatin in a manner distinct from siRNAs [107]. Piwi and Aubergine proteins exhibit “Slicer” activity when bound to piRNAs and cleave their piRNA's cognate RNA [128, 129]. Tudor domain proteins and arginine methylation of Piwi/Aubergine by the PRMT5 methyltransferase modulate Piwi/Aubergine association with piRNAs [130–133]. In addition, Piwi subfamily members may regulate the translation of germline transcripts [134, 135] and affect chromatin architecture to promote silencing [69, 136–138].

Analysis of the aphid genome sequence has revealed that the miRNA pathway has expanded in aphids [139]. *Drosophila* contains two Dicer genes, *Dicer1* and *Dicer2*, while mammals and *C. elegans* possess only one *Dicer* [140]. Jaubert-Possamai et al. [139] showed that the aphid genome, however, contains single copies of the *Dicer2* and *Ago2* siRNA pathway components and duplicates of *Pasha*, *Dicer1*, *Loquacious* and *Ago1* miRNA pathway genes relative to *Drosophila* (Figure 2). Aphid *Ago1b* and two of the four *Pasha* paralogs have undergone rapid evolution since duplication. The aphid *dcr-1a* and *dcr-1b* genes are lineage-specific duplications distinguished by a 44-amino acid insertion in the first RNAse III domain of DCR-1B. The aphid-specific duplication of Loquacious, a partner protein of Dicer1 that binds to precursor miRNAs and esiRNAs, complements the *Dicer1* duplication. These potential binding partners could form an array of complexes that regulate gene expression.

The identification of miRNAs encoded by the aphid genome firmly establishes the presence of active small RNA pathways in aphids [141]. Legeai et al. [141] used homology, deep sequencing, and predictive methods to converge on 149 pea aphid miRNAs, of which 55 are conserved among insects and 94 are thus far aphid-specific. Seventeen miRNAs showed differential expression among asexual, sexuals-producing asexual, and sexual females. Polyphenic locusts [142] and honeybees [143] also express small RNAs in morph-specific patterns. Aphid miRNAs can now be tested for their roles in aphid plasticity. As of yet, no aphid esiRNAs or piRNAs have been identified, but these small RNAs could be identified by prediction or by empirical methods.

The piRNA pathway also expanded in aphids. Within aphids, the *Piwi/Aubergine* subfamily has undergone extensive gene duplications, with eight *Piwi* paralogs and three *Ago3* paralogs found in the genome ([144], and Figure 2). Parallel to the expansion of aphid *Piwi/Aubergine* members, the aphid genome contains three PRMT5 methyltransferase paralogs (compared to one in *Drosophila*) [61], at least three Tudor-domain containing proteins and two copies of the HEN1 2′-OH RNA methyltransferase (D. G. Srinivasan, unpublished results). Similarly, in *C. elegans, *27 Argonaute family proteins have been identified—some without Slicer activity—that act in different aspects of the small RNA pathway [145]. This may be the case in aphids as aphid *Piwi* paralogs do show differential expression between different aphid morphs [144]. The high number of repetitive and mobile genetic elements in the aphid genome mirrors the expansion of the Argonaute protein family in aphids [42, 44, 146]. This suggests morph-specific regulation of transposons and mRNAs in a Piwi-dependent manner.

## 9. Current Hypotheses and Comparisons with Other Arthropod and Nonarthropod Systems

Most of what is known about the patterns and processes associated with DNA methylation come from studies in noninsect systems, primarily in mammals and plants. From these studies, a view emerged that methylation levels are high in CpG contexts, with transposons, other repeats, promoters, and gene bodies exhibiting methylation [78, 93, 147]. Promoter methylation is associated with a downregulation of transcription. More recent studies have shown that gene body methylation is ancestral to eukaryotes, but other methylation patterns, such as methylation of transposons, are taxon specific [148, 149].

Methylation in insects has traditionally been understudied due to the finding that *Drosophila melanogaster*, the most well-developed insect model, has almost no detectable DNA methylation [150]. It was therefore assumed that DNA methylation does not play an integral role in insect biology as it does in mammals and plants. Recent efforts have changed this impression. Whole-genome bisulfite sequencing of *Apis mellifera* [151] and *Bombyx mori* [91] has shown that insect genomes are, indeed, methylated. However, these studies have also shown that there are key differences between insect methylomes and vertebrate or plant methylomes. First, less than one percent of cytosines in insects is methylated compared with 20–80% in plants and mammals. Second, as mentioned above, insects exhibit variable numbers of each of the Dnmt enzymes. Third, methylation in insects is highest in gene bodies. And finally, transposable elements and other repetitive elements do not appear to be methylated at high levels. One pattern is shared among insects, plants, and mammals: genes with the highest and lowest expression levels show the least gene body methylation, while those with moderate levels of expression are the most highly methylated [68, 88, 148].

One intriguing idea that insect methylation studies have raised is the possibility that gene body methylation controls alternative splicing of transcripts. In fact, methylation in *A. mellifera* is enriched near alternatively spliced exons, and alternative transcripts of at least one gene are expressed in workers versus queens [68, 151]. Thus, methylation could control alternative splicing, with alternative transcripts being deployed to achieve alternative phenotypes. In general, because of their smaller genomes, accessibility as study organisms, and gene body methylation, insects may emerge as valuable systems for understanding the causes and consequences of DNA methylation [82].

How can DNA methylation be coupled to other epigenetic pathways in aphids? The measurement of relative methylation and accompanying chromatin states is a clear first step to test the connections between aphid gene duplications, gene expression, and chromatin structure. The interplay between chromatin modifications and DNA methylation may converge on differential expression and/or splicing of morph-specific genes. Additionally, small RNAs are expressed in morph-specific expression patterns in polyphenic locusts [142] and honeybees [143], and loss of *piwi* in *Drosophila* is associated with the loss of heterochromatic histone modifications and of HP1 association with chromatin in somatic cells [138]. Interestingly, the piRNA pathway in *Drosophila* has been associated with the suppression of phenotypic variation through the Hsp90 pathway [152] and with *de novo* DNA methylation of an imprinted locus in mice [153]. Additionally, *Drosophila* piRNAs can be epigenetically transmitted from mother to egg and affect the suppression of transposons in the next generation [154]. Identification, characterization, and correlation of small RNAs, DNA methylation, and chromatin structure to polyphenic aphid traits will help resolve the epigenetics underlying aphid life cycles.
